# Digital reconstruction of fragmented tooth remains in forensic context

**DOI:** 10.1080/20961790.2020.1737462

**Published:** 2020-04-21

**Authors:** Abraham Johnson, Gargi Jani, Joe Adserias Garriga, Astha Pandey

**Affiliations:** a Institute of Forensic Science, Gujarat Forensic Sciences University, Gandhinagar, India; b Department of Applied Forensic Sciences, Mercyhurst University, Erie, PA, USA

**Keywords:** Forensic sciences, forensic odontology, forensic reconstruction, three-dimensional surface scanning, three-dimensional printing

## Abstract

Forensic odontology majorly focuses on the identification of victims through the analyses of oral and para–oral structures. Exposure to high temperatures and trauma can occur in mass disasters and may lead to the fracturing and fragmentation of teeth. These fragments may become very fragile and easily damaged while handling. Conventional methodologies such as the use of transparent nail polish, hair spray, cyanoacrylate or adhesives have been used to stabilize the fragmented pieces. This study introduces a new and innovative digital technique that utilizes three-dimensional surface scanning (3DSS) and rapid prototyping techniques to reconstruct fractured portions of the teeth. The results of qualitative congruency analysis suggest that over all variance of morphological error (0.0526 ± 0.05) mm. These results imply that the reconstructed 3D model can be used for various morphometric analyses.

## Introduction

Forensic odontology is a branch of dentistry that involves using oral and para-oral structures of human remains such as teeth, mandibles, and maxillae as part of criminal investigation and identification in a variety of forms, especially for mass disasters and accidents where the human remains are decomposed, charred or skeletonized [1]. As per the INTERPOL guidelines, comparative dental analysis is one of the primary methods for human identification [2]. However, in mass disasters or in cases of trauma to the maxillofacial region, teeth may fracture. In some reported cases, fragmented bones and teeth have been found at the site of the incident as well as at the sites of mass disasters. In terms of fragmentation, reconstructive techniques can be applied. Transparent nail polish, hair spray, or cyanoacrylate/adhesives have been used to stabilize fragmented or fragile parts of teeth [3]. These reconstructed teeth may be re-articulated in their appropriate alveolar sockets and be used as additional points of comparison [4] for identification or investigation.

Three-dimensional (3D) scanning, computer aided design and computer aided manufacturing (CAD/CAM), and rapid prototyping [5] have taken conventional dentistry to the next level of treatment. In virtopsy, 3D imaging of human remains is a widely-accepted technique where the the principle of triangulation is used [6]. 3D imaging and printing is an emerging field in forensics that would help the forensic odontologists in the analyses of damaged dental remains and assist in case presentations. Reconstructive forensic refers to reconstruction and remodeling of fragmented and missing elements of the evidence thereby reconstructing the profile of evidence in question. Integrating reconstructive forensic odontology with digital manufacturing technologies would also be beneficial, particularly when handling of fragmented and burned dental remains could damage the specimens. To the best of our knowledge, 3D scanning and rapid prototyping techniques are not currently used for the reconstruction of fractured/damaged teeth. The study was conducted in the Laboratory of Forensic Odontology at Gujarat Forensic Sciences University (GFSU), Gujarat, India.

## Materials and method

### Study setting

This study was conducted in the Gujarat Forensic Sciences University (GFSU), Gujarat, India. The dental samples included 10 teeth (five maxillary central incisors, five maxillary lateral incisors) from the skeletal collections of Laboratory of Forensic Odontology, GFSU.

### Optical scanning

**Figure 1.**
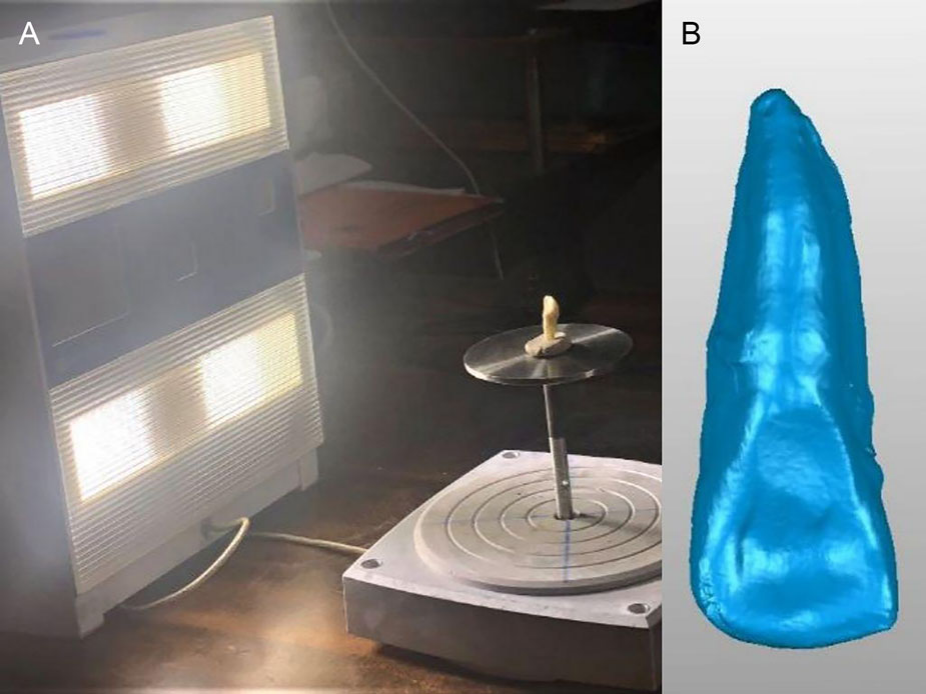
Laser scanning of the tooth specimen (A) and the 3D model of the scanned specimen (B).

A laser scanner (NextEngine 3D Laser Scanner, Santa Monica, CA, USA) with an accuracy of ± 0.040 µm (at Jet 3D Scan, Gujarat, India) was used to obtain digital images/data for all 10 samples. The resultant images provided a non-contact data collection from various angles covering the complete geometry of the scanned tooth. 3D-surface triangular mesh was obtained in Standard Tessellation Language (STL) format after assembling all the measurements using Geomagic Studio software (3D Systems, Rock Hill, SC, US) (Figure 1). The 3D models STL format) served as a reference model.

### Sample preparation

**Figure 2.**
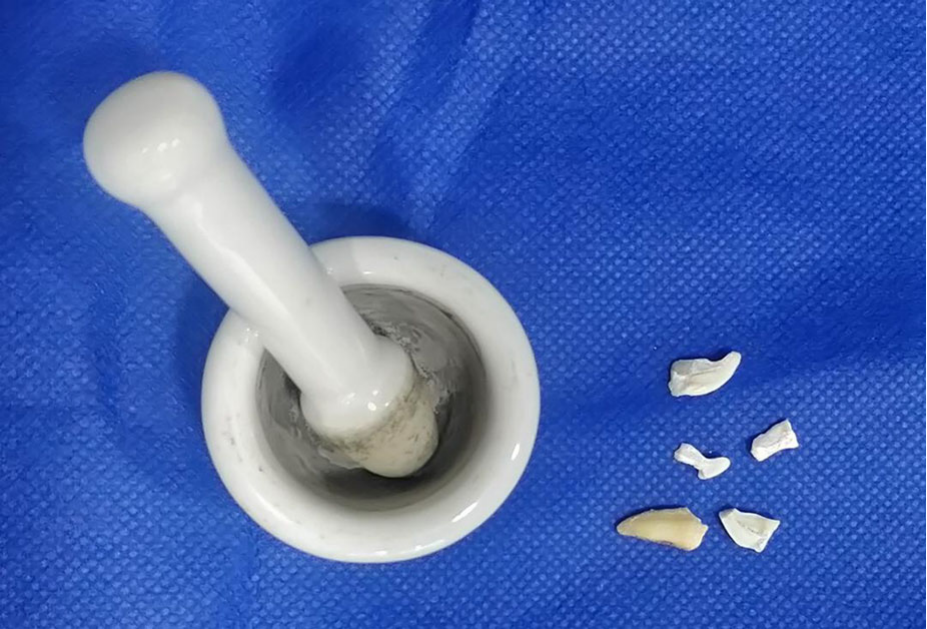
Fragmented tooth samples were obtained using a mortar and pestle.

A mortar and pestle were used to fracture all dental samples (Figure 2).

### 3D reconstruction procedure

**Figure 3.**
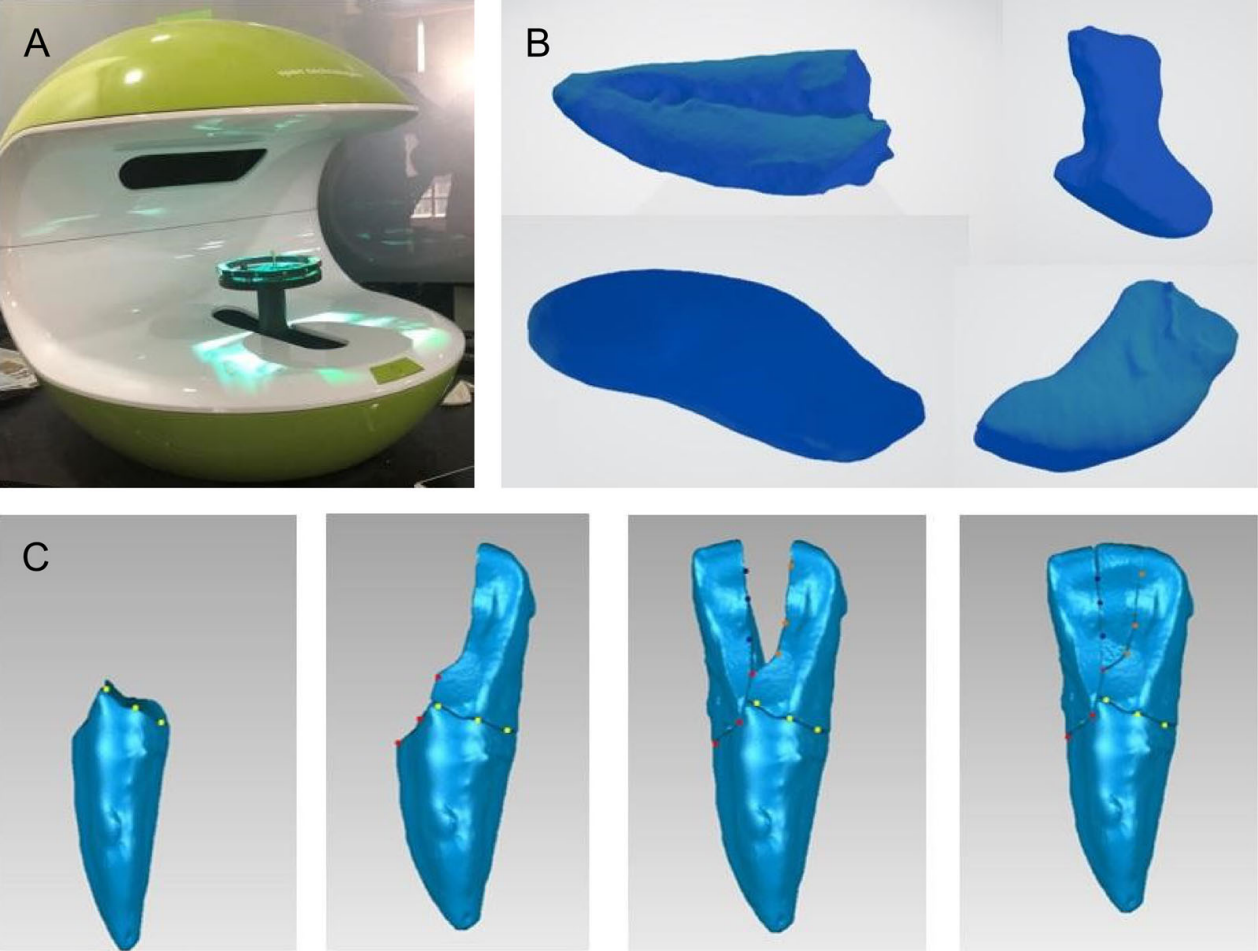
(A) Scanning of the fragmented tooth pieces using optical scanner. (B) 3D model obtained in Standard Tessellation Language (STL) format. (C) Multi-point registration performed manually, fragmented.

The fragmented tooth pieces were scanned individually using the structured light (Neway Dental Scanner, Open Technologies, Italy). These pieces were then aligned using Geomagic Studio software, as the alignment process is considered as the most important part for the entire workflow. A multi-point registration was performed manually, utilizing a set of few unique characteristic points on the surface of the models (Figure 3). The automatic matching process was initiated. This process used an algorithm that relies on minimizing the distance (mean square error) between two objects with the points on the crown and top of apices, as well as points determining the border of enamel and dentine.

**Figure 4.**
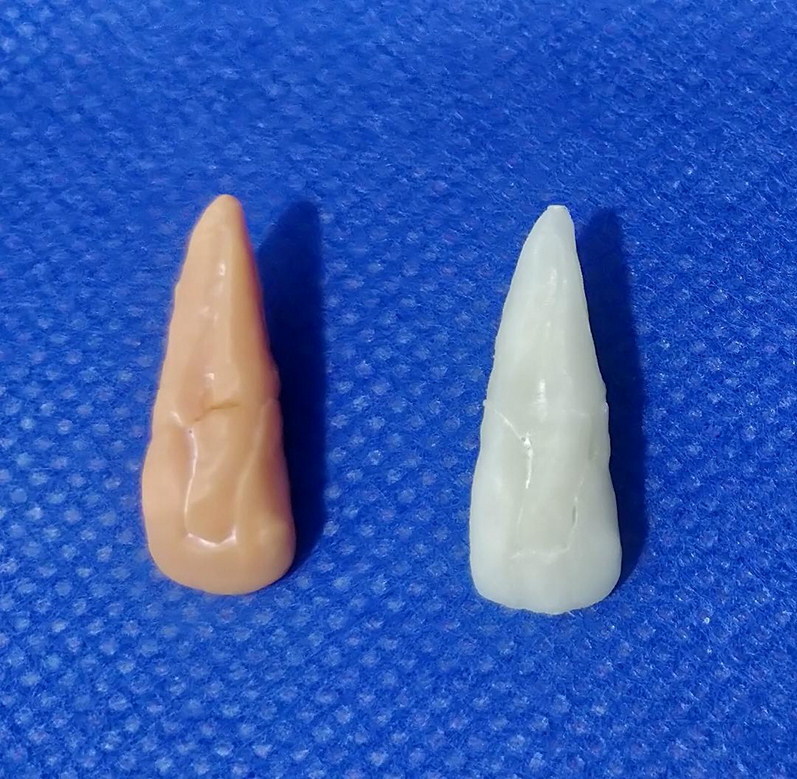
Models of reconstructed teeth which were printed using tooth printed by stereolithography technique (SLA) and tooth printed by fused deposition modelling technology (FDM) techniques (left to right, respectively).

The procedure for registering 3D objects used in this paper is commonly used in reverse engineering as used in the fields of mechanical engineering, software engineering and chemical engineering. Finally, the reconstructed teeth were converted into STL files and printed using poly lactic acid (PLA) material by fused deposition modelling (FDM) technology and photo polymerizing resin using the stereolithography (SLA) technique (Figure 4).

Further comparison was made between the reconstructed and reference models through superimposition of data. The comparison analysis was performed by an automatic algorithm implemented in Geomagic software, which is a standard tool into technical comparisons of 3D models (Computer Aided Design systems). The parameters for analysis between surfaces of the reconstructed 3D models and reference 3D models were maximum distance (minimum and maximum), average distance (minimum and maximum), and standard deviation (SD).

## Results

### Metric analysis

Various linear odontometric measurements of the teeth were obtained from the reference teeth and 3D printed replicas. These measurements were used to evaluate the accuracy of the reconstructed models (Table 1). The following measurements were taken using a digital sliding caliper (Aerospace Digimatic, New Delhi, India):

**Table 1.** Linear odontometric measurements of the reference teeth and 3D printed replicas to evaluate.

		Maxillary central incisors					Maxillary lateral incisors				
Crown-root dimensions (mm)		SP:1	SP:2	SP:3	SP:4	SP:5	SP:1	SP:2	SP:3	SP:4	SP:5
Crown length											
	N	11.71	11.15	12.40	10.95	11.21	9.10	9.86	9.79	8.51	8.43
	SLA	11.44	11.01	12.09	10.65	11.17	9.12	9.65	9.67	8.51	8.14
	FDM	11.49	11.06	12.27	10.21	11.05	9.07	9.77	9.58	8.76	8.35
Mesio-distal width at incisal edge											
	N	9.29	8.74	9.34	8.36	8.87	5.20	6.26	6.30	6.35	6.87
	SLA	9.30	8.68	9.29	8.23	8.78	5.13	6.24	6.32	6.29	6.66
	FDM	9.25	8.54	9.11	8.19	8.65	5.21	6.27	6.24	6.21	6.54
Mesio-distal width at cervix											
	N	7.67	6.92	5.73	5.91	6.12	4.80	4.44	5.18	4.47	4.69
	SLA	7.64	6.84	5.45	5.47	6.23	4.76	4.34	5.12	4.45	4.56
	FDM	7.64	6.67	5.21	5.34	6.11	4.83	4.43	5.21	4.32	4.72
Bucco-lingual width at incisal edge											
	N	4.34	2.28	2.47	2.07	3.18	2.15	1.38	2.12	2.25	2.43
	SLA	3.76	2.12	2.23	2.11	3.16	2.02	1.31	2.10	2.21	2.32
	FDM	4.02	2.34	2.43	2.12	3.21	2.10	1.24	2.09	2.16	2.41
Bucco-lingual width at cingulum											
	N	6.67	7.46	7.07	6.87	6.91	6.09	5.49	6.02	6.25	6.34
	SLA	6.63	7.34	7.01	6.65	6.85	6.04	5.34	6.04	6.19	6.21
	FDM	6.68	7.24	7.12	6.66	6.87	6.02	5.26	6.10	6.28	6.11
Root length											
	N	15.88	13.10	13.25	12.54	13.54	12.19	12.12	12.20	13.35	13.34
	SLA	15.33	13.12	13.22	12.50	13.28	12.08	12.12	12.24	13.36	13.38
	FDM	15.29	13.09	13.19	12.47	13.34	12.10	12.07	12.10	13.12	13.38

SP: specimen; N: natural tooth; SLA: tooth printed by stereolithography technique; FDM: tooth printed by fused deposition modelling technology.

1. Crown length (CL)—Maximum distance from the incisal edge to the cervical line;
2. Mesio-distal width at incisal edge (MDI)—Maximum distance from the mesial margin to distal margin at the incisal aspect;
3. Mesio-distal width at cervix (MDC)—Maximum distance from the mesial margin to distal margin at the cervical aspect;
4. Bucco-lingual width at incisal edge (BLI)—Maximum distance from the buccal aspect to the lingual aspect at incisal edge;
5. Bucco-lingual width at cingulum (BLC)—Maximum distance from the buccal aspect to the lingual aspect at the cervix; and,
6. Root length (RL)—Maximum distance from the cervix at buccal aspect to the apex.

### 3D digital analysis

**Figure 5.**
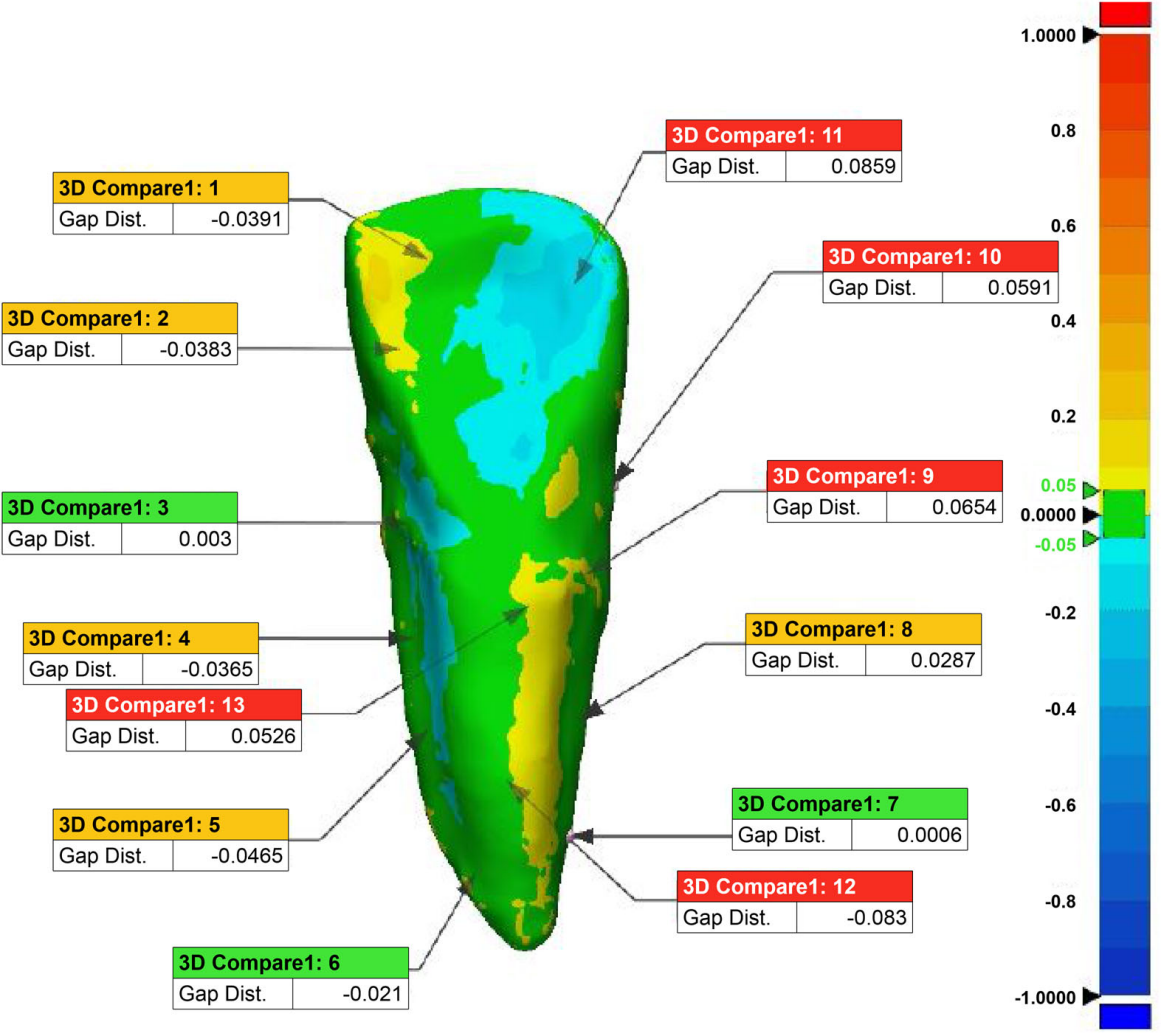
Qualitative congruency analysis performed on images of a reference tooth and a reconstructed tooth specimen.

The utilization of colored images allowed for a qualitative congruency analysis between reference teeth and reconstructed teeth (Figure 5). The maximum error range was set between −0.05 mm and +0.05 mm, which is acceptable in craniometrics. The areas of positive error are represented by the yellow and red regions, and the areas of negative error are represented by the blue regions. Areas where the error is near zero are represented by green regions. The mean ± SD of the RMS values is (0.0526 ± 0.05) mm, implying the overall level of variance of morphological error 0.0526 ± 0.05. The average value and variance are represented as 0.0005 mm and 0.0028 mm respectively. These results imply that the reconstructed 3D model can be used for various morphometric analyses with minimal variation from the actual specimen.

## Discussion

Three-dimensional surface scanning (3DSS) can collect data from various directions/angles without physically handling the specimen/object [7]. 3D printing is an advanced technology that models scanned data based on the object placed within the device, and controls the precise 3D placement of the printed material in order to create a model almost identical to the original object [7]. One of the earliest medical modelling applications involved the 3D printing of anatomical study models [8]. 3D printing is currently being used in the biomedical sciences, and the methods regarding the reconstruction of scanned images/objects are a source of continual research [7]. The combination of 3DSS and 3D printing technologies can be used in forensic practice to reconstruct the fragmented and burned remains. This would most certainly be a field of interest for use by many forensic practitioners [9].

In situations of mass disasters, the literature mentions the use of conventional methods and techniques of using glue, cyanoacrylate cement, and hair spray to stabilize and reconstruct the fragmented bones [9] and tooth pieces [3,4]. For individualization and identification, teeth are one of the most important methods, hence the reconstruction of fragmented teeth would be of great benefit. However, by using adhesive or cement the brittle dental remains may further fragment/fracture due to improper handling or manual pressure. To overcome these limitations the present study was designed to reconstruct the fragmented remains using digital scanning and manufacturing techniques.

With the advancement in technology, the 3D surface scanning has proved to give accurate reproducibility [10]. This precision is reflected by an accuracy range of ±0.05 mm for the reconstructed tooth. The morphometric and digital analysis of the reconstructed tooth using this 3D technique can be an aid in morphological and metric analyses which may lead to identification. In case of availability of the skeletal remains, the reconstructed tooth can be placed in the socket and the accuracy of the placement can be assessed using radiovisuography. In presence of antemortem data, the reconstructed tooth can aid in comparative identification. Additionally, teeth are a good source of genetic material for identification [7]. DNA can be extracted from enamel, dentin and pulp [11, 12]. However, in the process of extracting DNA for genetic analysis, the tooth pieces may be irreversibly destroyed [7]. Hence, if teeth are reconstructed digitally, the fragmented pieces may be used for DNA extraction and the reconstructed 3D printed tooth may be used for further analysis and presentation in courtroom. The printed tooth models can further act as a tool for metric analysis aiding in sex determination and age estimation. Also, the non-metric traits can be assessed and assist in determining population differentiation [13]. Apart from comparative identification, this may also help in reconstructive identification by determining the shape, thickness, and position of lips in cases involving anterior teeth and establishing occlusion in case of posterior teeth which shall aid in forensic facial reconstruction/approximation without further damaging the remains [14,15].

Limitations of this approach includes the requirement for a special setup and expert intervention, high cost, material selection for printing, need for accurate/precision scanning device, and the inability to assess unrestored and restored teeth. Although with time and improved technology, this technique may become more economical and widely used. Further research on addressing the unrestored/restored teeth is planned and will be addressed in the future.

## Conclusion

Teeth fragmented into multiple pieces may be reconstructed using 3DSS and 3D printing technology with high precision. The model/reconstructed tooth may aid in various forensic investigations as well as in forensic facial reconstruction, where the fragments are too fragile to handle by an analyst or when the fragmented pieces may be removed and used for DNA extraction/analysis. The digital manufacturing methods produced an accuracy range of ±0.05 mm. Standardizing the method of reconstruction of teeth owing to accuracy of scanning and printing devices in the forensic field is still required. The process of fragmented tooth reconstruction shall be continued in further studies.
